# Toward a New Understanding of Graphene Oxide Photolysis: The Role of Photoreduction in Degradation Pathway

**DOI:** 10.1002/advs.202414716

**Published:** 2025-02-03

**Authors:** Yuchen Yang, Nanzhi Zheng, Chen Ma, Silong Chen, Wenhua Chen, Guohua Chen

**Affiliations:** ^1^ College of Materials Science and Engineering Graphene Powder & Composite Materials Research Center of Fujian Province The Xiamen Key Laboratory of Polymers & Electronic Materials Huaqiao University Xiamen 361021 P. R. China

**Keywords:** graphene oxide, photolysis, photoreduction, photo‐transformation, sunlight irradiation

## Abstract

Graphene oxide (GO) is developed in various applications owing to its fascinating physicochemical properties. However, the weak photostability always leads to inevitable photolysis of GO during the use, storage, and application. Indirect photolysis has a significant impact on the structure of GO via causing fragmentation and degradation, while the pathway can be divided into two stages. In the early stage, photoreduction is the dominant reaction to generate porous reduction GO (PrGO). Then H_2_O_2_ breaks PrGO into fragments, and eventually, the fragmented GO is converted into CO_2_ by OH radicals. The generation of porous structures in early photoreduction is a crucial premise for the subsequent photodegradation, while GO flakes without porous structure cannot be broken by H_2_O_2_ and OH. In this work, a deep insight into the indirect photolysis pathway and the committed step is provided, which may bring some advanced ideas for enhancing GO stability in practical application.

## Graphical abstract

The indirect photolysis of GO can be divided into two stages. In the early stage, photoreduction is the key step to generate PrGO, and then can be cut and oxidized into Nano GO and CO_2_. GO cannot be photolysis without a porous structure generated by photoreduction.

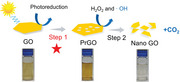

## 1 Introduction

Graphene oxide (GO), a fascinating derivative of graphene,^[^ ^1^, ^2^, ^3^ ^]^ displays the myriad applications in water purification,^[^ ^4^, ^5^ ^]^ photocatalysts,^[^ ^6^ ^]^ electrocatalysts,^[^ ^7^ ^]^ and nanofillers in various composite materials.^[^ ^8^, ^9^, ^10^, ^11^, ^12^ ^]^ In contrast to graphene, GO has abundant oxygen‐containing functional groups (OFGs), including carboxyl groups on the edges and hydroxyl and epoxy groups on the surface.^[^ ^3^, ^13^ ^]^ OFGs disrupt the hybrid network of sp^2^ carbon and make GO electrically insulating, while GO is endowed with water solubility and solution processability.^[^ ^14^, ^15^, ^16^ ^]^ More importantly, GO shows photochemical reactivity due to the destruction of the zero band gap caused by OFGs.^[^ ^17^, ^18^ ^]^ As a reactive nanoparticle, GO is difficult to maintain its pristine form during daily use and storage.^[^ ^19^, ^20^, ^21^ ^]^ Solar irradiation is the most common factor that can induce and affect the transformation of GO, thus changing the chemical characteristics as well as destroying the morphology of pristine GO.^[^ ^22^, ^23^ ^]^

As is well known, the most important photoreactions of GO are photoreduction and indirect photolysis.^[^ ^24^, ^25^, ^26^, ^27^, ^28^ ^]^ During the photoreduction process, solar irradiation generates electron‐hole pairs, transforming the GO flakes into porous structures with partial reduction.^[^ ^29^, ^30^ ^]^ The indirect photolysis of GO is generally considered to be caused by OH radicals with a strong oxidation potential (2.8 V).^[^ ^31^, ^32^ ^]^ GO flakes will be converted into low molecular weight species (LMWs) and fragments during the indirect photolysis process.^[^ ^24^, ^33^, ^34^, ^35^ ^]^ In some previous studies, Bai et al.^[^ ^33^ ^]^ used the photo‐Fenton reaction to investigate the potential mechanism of GO degradation. They determined that intermediate oxidation products comprised LMW polycyclic aromatic hydrocarbons, and the final product was dominated by graphene quantum dots (GQDs). Zhang et al.^[^ ^34^ ^]^ speculated on the detailed mechanism of OH attacking the surface functional groups of GO. In Duan's work.^[^ ^36^ ^]^ OH radicals cannot only react with OFGs on the surface of GO but also with aromatic domain area through electrophilic. According to Shams's research,^[^ ^37^ ^]^ hydroxyl and epoxy functional groups were the most easily photolysis, while decreased functional groups could weaken the photolysis capacity. Subsequently, Shams et al.^[^ ^38^ ^]^ extended their research to natural aquatic environments and discussed the photolysis of GO with different oxidation levels in Columbia River water. Radich's^[^ ^39^ ^]^ study showed that OH radicals could attack rGO and then cause more polyaromatic hydrocarbons (PAHs) compounds as the platform for photocatalysis. Todorova et al.^[^ ^26^ ^]^ proposed that the instability of GO under irradiation should be considered when using solar reduction to prepare reduction GO. Zhang et al,^[^ ^40^ ^]^ report that when the GO aqueous was exposed to air for long‐time irradiation (such as 2 years), the electron‐donating active components of GO facilitates the conversion of O_2_ to O_2_^−^, which are subsequently converted to H_2_O_2_, ultimately leading to the formation of •OH, resulting in the degradation of GO into small fragment molecules.

To date, numerous investigations have been conducted on the photolysis of GO under OH. However, the generation of OH is usually accompanied by irradiation, which can induce photoreduction and influence the GO photolysis. Such dual factors of OH radical and irradiation on GO reduction are unknown due to the insufficient understanding of the GO phototransformation process.

In this study, GO/H_2_O_2_ systems were used to simulate indirect photolysis. We made a significant discovery of the indispensable role of photoreduction in this process. This process leads to the formation of pores as GO indirect phototransformation. Our finding supported the following pathway for GO fragmentation: light irradiation induced GO bandgap transition to generate porous structures through h^+^ oxidation and then react with H_2_O_2_ to transform into fragments. When h^+^ is quenched, the fragmentation process cannot proceed. This study reveals the unique relationship between photoreduction and the degradation of graphene oxide nanomaterials.

## 2 Results and Discussion

### 2.1 Structural Evolution of GO During Photolysis

The morphology and chemical composition of synthesized GO were systematically studied and related results were shown in Figures S1 and S2, Supporting Information. It could be indicated that GO prepared by the modified Hummers method shows the single‐layer structure with abundant oxygen functional groups and was readily dispersed in DI water.

**Figure 1:**
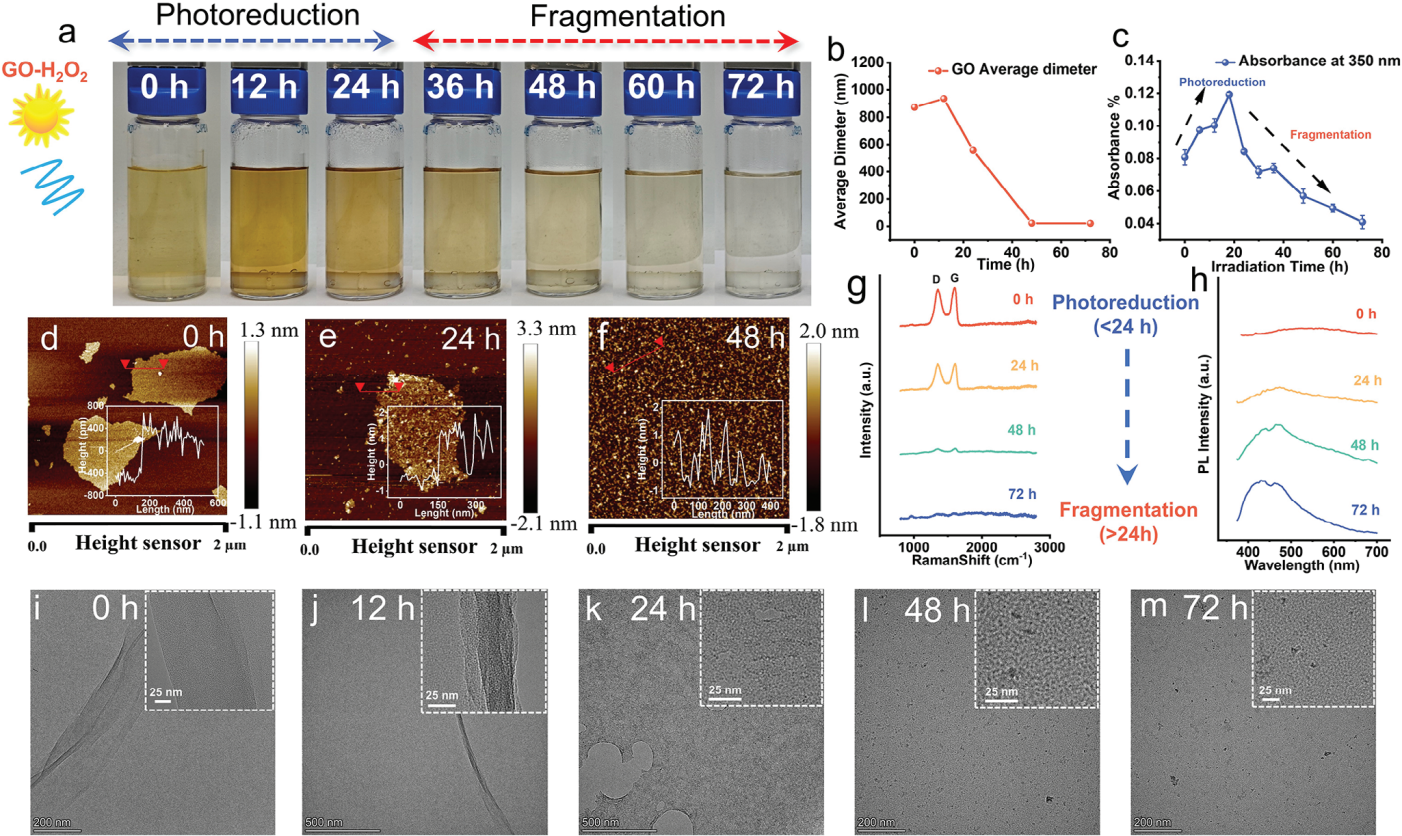
Phototransformation of GO/H_2_O_2_ system under simulated sunlight irradiation. a) Photographs showing the appearance changes of the sample by extending the irradiation time. b) average diameter. c) absorbance at 350 nm. d–f) AFM images. g) Raman spectra. and h) Photoluminescence patterns of GO for different irradiation times. i–m) TEM and HRTEM images.

As shown in the digital photos in **Figure** **1**a, the color of the GO/H_2_O_2_ mixture was deepened after the first 24 h irradiation and gradually bleached after 72 h, while the mixture eventually transformed into a colorless solution. This result indicated a significant structural transformation of GO. We observed absorbance at 350 nm,^[^ ^35^ ^]^ and the changed curve was plotted in Figure 1b. The absorbance increased during the first 18 h of irradiation and then decreased, while the value in 24 h irradiation was higher than the original absorbance. Apparently, the changes in absorbance at 350 nm are consistent with the color change of the sample (the same trend of absorbance at the range of 300 – 800 nm can be found in Figure S3, Supporting Information).

The AFM image shows the entire process of surface structure change in GO (Figure 1d–f). With increasing photolysis time, the surface structure of GO layers transformed from intact to porous and then fractured. After 48 h irradiation, all GO layers turned into fragments with a diameter of ≈20 nm, which was considered as Nano‐GO. The size distribution analysis of AFM images (Figure 1c) also shows the conversion of a large layer into a small layer, with diameters all less than 40 nm after 48 h irradiation. In the AFM image of the sample at 72 h irradiation (Figure S4, Supporting Information), there is no significant difference in structure after 48 h, indicating that the main structural transformation occurred at 48 h.

This nonlinear change in color indicates that photolysis is not a unique reaction, while the initial increase and then decrease in absorbance and GO become porous were also suggests that photoreduction and photolysis occur concurrently. At the same time, we also characterized the direct photolysis of GO. During the irradiation time increased, the color of the solution deepens and absorbance increases (Figure S5, Supporting Information). The GO flake transformed into a porous structure via irradiation, which can be directly observed by AFM/TEM images (Figure S6, Supporting Information). This transformation is similar to that found in the early stage of indirect photolysis.

To further investigate the structural transformation in photolysis, we introduce synchronous verification of the photolysis process using Raman and photoluminescence spectra. As shown in the Raman spectra in Figure 1g, a fixed laser wavelength at 532 nm was used to detect the sample. The intensity ratio between the characteristic D band (≈1350 cm^−1^) and G band (≈1600 cm^−1^) or *I*_D_/*I*_G_ provided information about the disorder in the graphitic lattice of the GO sheets.^[^ ^41^ ^]^ After 24 h of indirect photolysis, the *I*_D_/*I*_G_ increased from 0.92 to 1.02. This increase could be due to the h^+^ generated by bandgap transitions in the early stage photoreduction. It in turn formed more defect sites on the graphite lattice of GO, also consistent with the photoreduction result previously reported.^[^ ^26^ ^]^ Subsequently, *I*_D_/*I*_G_ decreased to 0.83 at 48 h, which could be attributed to the formation of more amorphous carbon structures or sp^2^ non‐crystalline carbon phase by high defect density as reported previously.^[^ ^42^, ^43^ ^]^ This similar type of low *I*_D_/*I*_G_ was reported for GQDs synthesized through photo‐Fenton reaction, which involved the generation of OH radicals.^[^ ^44^ ^]^ However, the absolute intensity of the characteristic broad D and G bands were remarkably diminished after 24 h. Finally, after 72 h indirect photolysis, the D and G bands disappeared. This is due to the significant fragmentation or degradation of GO,^[^ ^42^, ^45^, ^46^ ^]^ which was further confirmed by AFM/TEM images at 72 h (Figure 1m; Figure S4, Supporting Information)

To investigate the degradation process further, the photoluminescence spectra were used to track the sample (Ex wavelength at 365 nm).^[^ ^35^ ^]^ As shown in Figure 1h, the sample demonstrated a broad peak centered on 550 nm at the beginning of irradiation.^[^ ^47^ ^]^ At 24 h, the emission peak was concentrated at ≈450 nm. After 48 h irradiation, the peak was significantly enhanced by 285% compared to 24 h. AT 72 h, the emission peak was slightly blue shift to 430 nm.

The generation of strong fluorescence at 430–450 nm provides evidence of the fragmentation of GO flakes. In Figure 1e, as the average diameter of GO decreased to ≈20 nm, the PL emission was also generated at ≈450 nm. This could be attributed to Nano‐GO's character being similar to GQDs‐like compounds. It could cause a fluorescence emission peak at ≈450 nm.^[^ ^48^, ^49^ ^]^ In fact, many recent studies have shown that physical or chemical treatments of graphene or graphene oxide produce strong fluorescent GQDs, which are attributed to the quantum confinement or edge effects generated by nanoscale GQDs. In addition, the PL peak has a slightly blue shift at 72 h irradiation. This could be attributed to the Nano‐GO size being slightly smaller than 48 h irradiation, which is consistent with quantum effects.^[^ ^51^ ^]^ This increase in fluorescence provides evidence that GO is broken down into fragmented products (GQDs or Nano‐GO).

TEM and HRTEM images were also used to analyze the process of photolysis. The pristine GO shows a completed flake and distinct edges (Figure 1i). When irradiation at 12 h, the flake edges show a multilayer structure, this could be attributed to photoreduction‐induced GO flake stacking (Figure 1j). After 48 h irradiation, the GO flakes generated many holes and became porous (Figure 1k). The GO layer turns into Nano‐GO at 48 h (Figure 1l), and 72 h Nano‐GO size is slightly smaller than 48 h (Figure 1m). These results support the evolution observed in the PL and Raman spectra.

Overall, AFM/TEM images, PL, Raman, and UV–vis absorbance spectra all confirmed that the photoreduction occurred in GO photolysis early stage, then degradation into Nano‐GO.

### 2.2 The Role of Radical and Hole for GO Photolysis

According to previous reports, photoreduction is the generation of hole‐electron pairs by the band gap transition of GO itself. The strong oxidation of h^+^ easily causes defects in carbon and then turns into CO_2_, e^−^ combined with OFGs induced the reduction. Eventually, GO layers were porous and reduced.^[^ ^29^, ^30^, ^50^ ^]^ Photolysis of GO is considered to be caused by the strong oxidation of OH, which could react with OFGs on the GO surface and break GO flakes into fragments.^[^ ^33^, ^34^, ^44^ ^]^ Herein, we have confirmed that photoreduction occurred in the early stage of photolysis. To investigate the impact of photoreduction in photolysis and the relationship between these two kinds of photoreactions. We used IPA as the OH scavengers to prohibit its degradation of GO and EDTA as the holes scavengers to prevent the generation of h^+^ and porous structure caused by photoreduction in photolysis.

**Figure 2:**
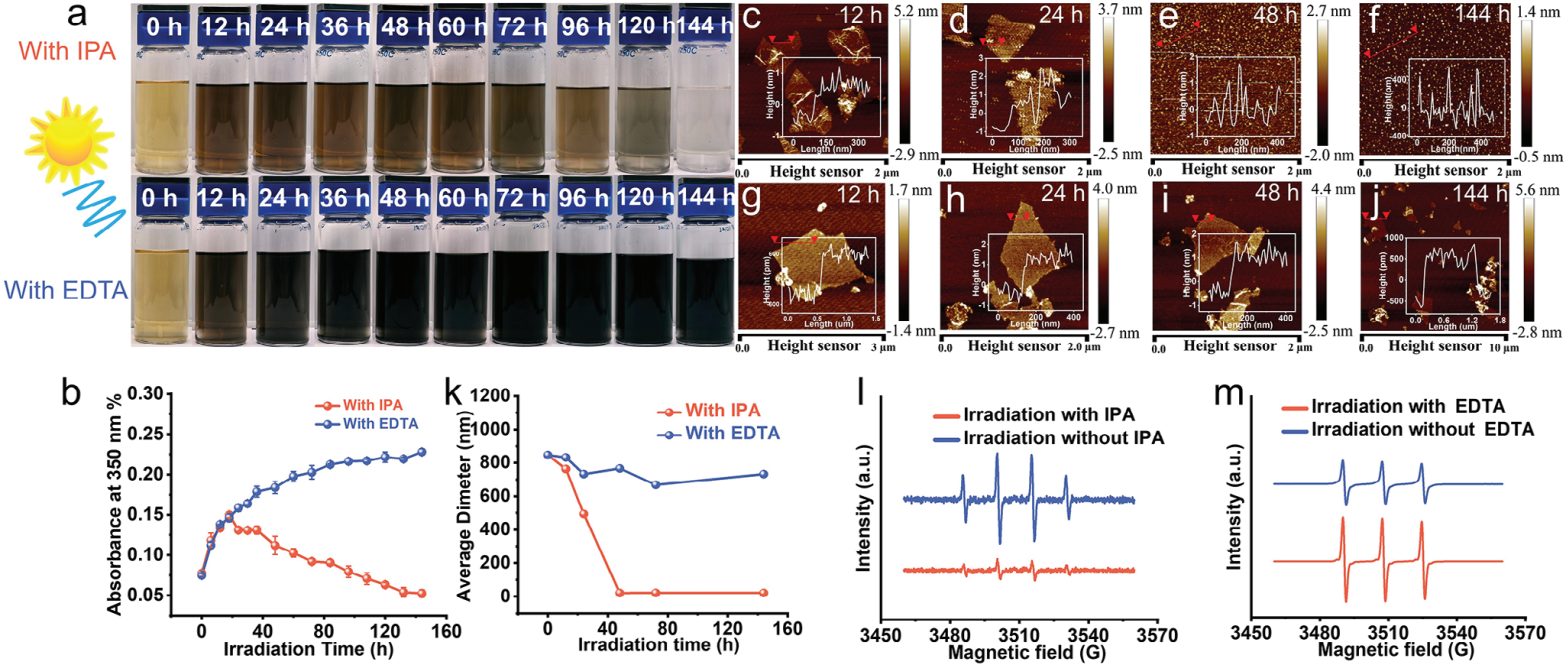
Indirect phototransformation of GO containing different scavengers (IPA OH scavenger and EDTA h+ scavenger) during simulated sunlight irradiation. Showing evolutions of, a) Sample color. b) UV–vis absorbance at 350 nm. c–f) AFM image of sample surface structure with IPA. g–j) AFM image of sample surface structure with EDTA. k) Size distribution analyses. l) ESR signal of DMPO‐OH. m) ESR signal of TEMPO.

**Figure** **2**a shows the color changes during GO photolysis irradiation after adding different scavengers. It can be clearly seen that after the addition of IPA, the color of the GO solution first deepened and then bleached. Although the addition of IPA delayed the decreasing rate of absorbance, the sample still transformed into colorless after 144 h irradiation. The absorbance change is also consistent with color, increased at an early stage and then decreased (Figure 2b). As shown in Figure 2c–f, the AFM image further shows the structural transformation of GO after adding IPA. We can observe that the sample still exhibits a process similar to photolysis. The GO flakes become porous in the early stage and then converted to Nano‐GO after 48 h irradiation. Figure 2k shows the GO average diameter size on multiple AFM data in photolysis. After 48 h irradiation, the average diameter was all distributed at ≈20 nm, indicating the addition of IPA did not prevent the photolysis. Meanwhile, we used ESR to detect the effect of IPA as OH scavengers. DMPO was used as a free radical trap. It can be observed that after adding IPA, the signal of DMPO‐OH was significantly weakened compared to before, indicating that OH radicals were captured by IPA effectively (Figure 1, 2). In a word, the addition of IPA can effectively capture OH radicals but has no significant effect on the photolysis process, indicating that photolysis could not be dominated by OH. The bleaching time in the IPA/GO/H_2_O_2_ system was significantly prolonged, which can be attributed to the OH being captured, decreasing attack on the *π*–*π* conjugated region on GO, resulting in a slow decrease in absorbance.

For the EDTA/GO/H_2_O_2_ system, the color of the solution continued to deepen during irradiation, which is consistent with the 350 nm absorbance change in Figure 2b (The absorbance changes at wavelengths of 300–800 nm were shown in Figure S7, Supporting Information). AFM images showed the GO layer structure in Figure 2g–j, it remains intact throughout phototransformation without generation of porous structure and Nano‐GO (some prominent parts in the AFM image are EDTA has not been completely removed). The average diameter of GO layers also supports these results. It is only slightly decreased compared to the initial value (Figure 2k). ESR results verify the quenching effect of EDTA on h^+^. It can be found that after adding EDTA, the TEMPO signal is stronger than before (Figure 2m), because EDTA effectively quenches h^+^, indicating that EDTA as h^+^ scavenger is effective in this system.^[^ ^52^ ^]^

**Figure 3:**
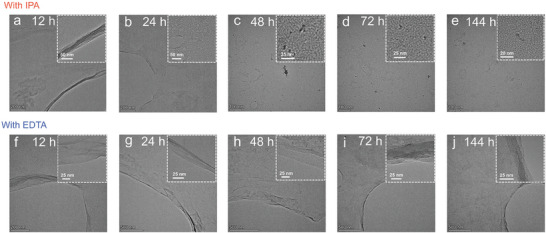
TEM and HRTEM images of GO indirect photolysis with scavengers. a–e) with OH radicals scavengers IPA. f–j) with h^+^ scavengers EDTA.

TEM and HRTEM were used to analyze sample transformation during irradiation. When IPA was added as OH radical scavengers, The evolution of GO flakes was from intact to porous. After 48 h irradiation, no complete layer could be observed, all flakes had turned into Nano‐GO (**Figure** **3**a–e). These changes are similar to those observed during indirect photolysis, indicating that IPA has no effect on GO photolysis. While after adding EDTA as h^+^ scavengers, EDS mapping shows the signal of nitrogen on GO layers (Figure S8, Supporting Information), and GO flakes remained intact without becoming porous or forming Nano‐GO through photolysis (Figure 3f–j). As the irradiation time increased, the edges of GO began to stack. This could be attributed to the restoration of conjugated regions, which induced the tacking. TEM analysis was also confirmed with AFM results, showing that EDTA could prevent GO layers from becoming porous and fragmented.

**Figure 4:**
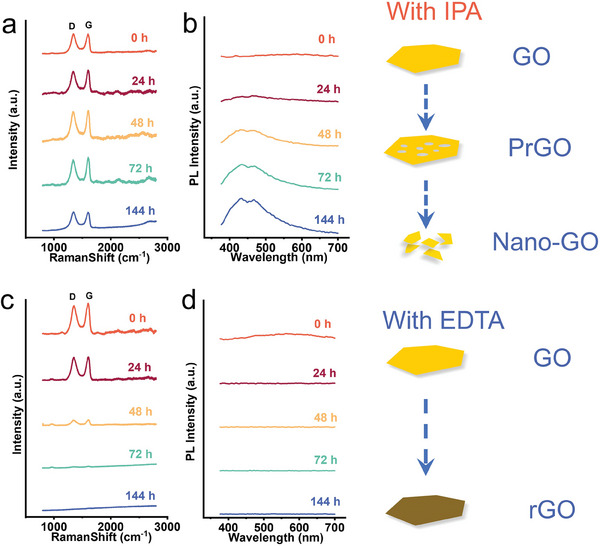
Changes in the structure and flake size upon different scavengers during photolysis. a,b) Raman and Photoluminescence Spectra with IPA. c,d) Raman and Photoluminescence Spectra with EDTA.

Raman spectroscopy and photoluminescence were also used to track the structural changes of GO during the indirect phototransformation process. As shown in **Figure** **4**a,b, in the presence of IPA as OH scavengers, the Raman spectra intensity of the D and G bands significantly decreased at 48 h and disappeared after 72 h. In PL spectra, with the extension of photolysis time, a PL emission peak centered at 450 nm generated and gradually blue shifts. These results indicated the structural changes of GO flakes are consistent with AFM and TEM images. It becomes porous in the early stage and fragmented into Nano‐GO. In the presence of EDTA as a h^+^ scavenger, the intensity of the D and G band intensity in Raman spectra remained unchanged, and the characteristic D and G bands of GO could still be displayed after 144 h. In PL spectra, the sample only exhibited original PL after irradiation, and without emission peak was generated throughout the process, especially at 450 nm, indicating that the GO layer did not fragment to Nano‐GO with PL characteristics, the loss of intrinsic emission can be attributed to the photogenerated electron caused reduction.

Overall, OH scavenger (IPA) has no effect on the process of GO photolysis. GO still became porous and then turned into Nano‐GO and sample absorbance increased in the early stage and then decreased. While h^+^ scavenger (EDTA) can effectively prevent photolysis, the GO layer remains intact throughout the whole process and absorbance continuously rises. Consequently, we concluded that the strong oxidizing nature of OH was not the main reason for the photolysis. The photoreduction generated h^+^ probably dominated the whole photolysis process. Based on this, we speculate that the photoreduction‐induced h^+^ caused formation of porous structures is the main reason for GO photolysis, rather than the strong oxidizing nature of OH leading to layer breakage.

### 2.3 Kinetic Analysis of Photolysis

To quantitatively elucidate each process of photolysis, we established a model based on UV–vis absorbance changes to analyze the whole process. The indirect photolysis of GO could be described as the following steps:

(1) $$H_{2}O_{2}\overset{hv}{\to }2\cdot \mathrm{OH}$$

(2) $$GO\overset{hv}{\to }PrGO+CO_{2}+H_{2}O$$

(3) $$PrGO+H_{2}O_{2}+\cdot OH\to NanoGO+CO_{2}$$

Equation (2) could be further divided into the following steps:

(4) $$GO\overset{hv}{\to }h^{+}+e^{-}$$

(5) $$GO+h^{+}\to PGO+CO_{2}$$

(6) $$GO+e^{-}\to rGO+H_{2}O$$

Equation (1) occurred throughout the entire photolysis process. Equation (2) mainly occurred in the early stage of photolysis which induced an absorbance increase. After irradiation, GO generates PrGO and CO_2_, while H_2_O_2_ and OH radicals further convert it into Nano‐GO and CO_2_ (Equation 3). Equation (3) is a fast process and results in a decrease in absorbance. Additionally, OH radicals could also attack GO *π*‐*π* conjugation region‐induced absorbance decreases. Based on the above results, we focus on the absorbance changes during GO photolysis. UV–Vis absorbance spectra were measured to analyze and quantify each photo‐driven process.

**Figure 5:**
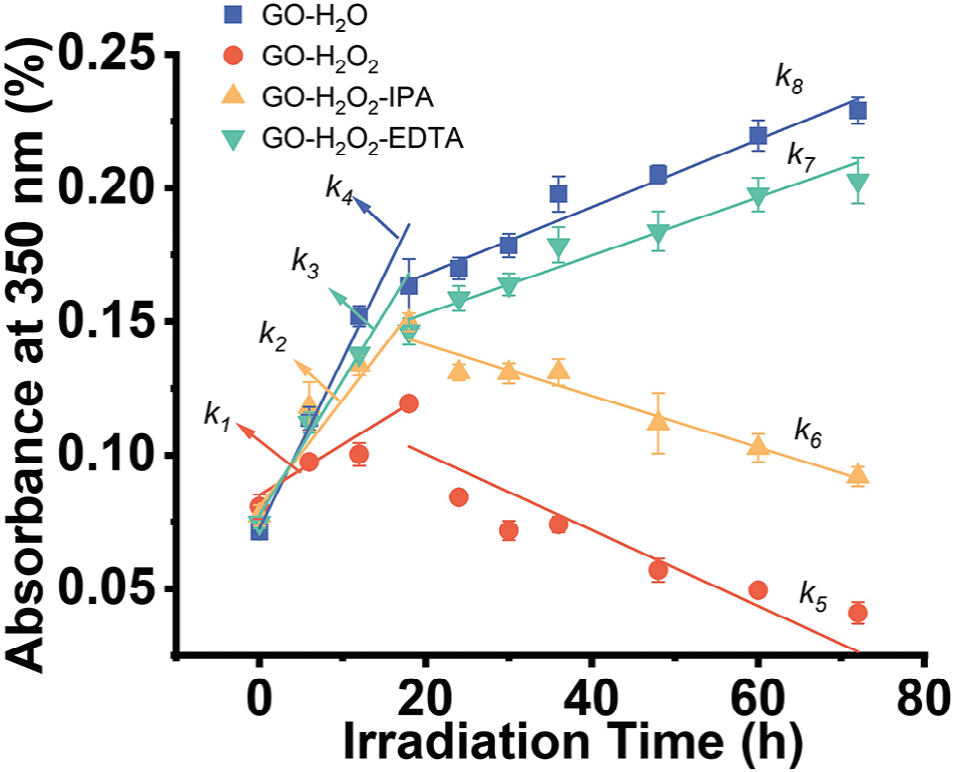
Two‐stage pseudo‐first‐order kinetics of absorbance evolution under indirect and indirect with scavengers phototransformation of GO.

In **Figure** **5**, we measured the absorbance change during direct photolysis, indirect photolysis, and indirect photolysis with OH scavengers and h^+^ scavengers. In the indirect photolysis early stage, direct photolysis (Equation 2) dominates the process‐induced absorbance increased, but due to OH generated via Equation (1) attack GO conjugated region. Therefore, the rate constant *k_1_* is slightly lower than direct photolysis in aqueous solution *k_4_*.

When the OH radicals were quenched, we observed that the direct photolysis rate became higher (*k_2_* > *k_1_*) in the early stage. This could be attributed to the exclusion of the reaction between OH and GO. When h^+^ were quenched, according to Le Chatelier's principle, Equation (4) could generate more e^−^, which induced Equation (6) to accelerate. Hence, although OH radicals can still attack GO, the photoreduction rate remains higher than in the early stage of indirect photolysis (*k_3_* > *k_1_*). These results indicate that holes and electrons dominate the early stage of photoreduction.

After Equation (2) generates PrGO in the early stage, Equation (3) becomes the dominant process for degradation, leading to a decrease in absorbance. *k_5_* represents the degradation rate in indirect photolysis, where the absorbance decreased, so *k_5_* < 0. After adding OH scavengers, there was a slight delay in the degradation rate (*k_5_* > *k_6_*). However, when h^+^ scavengers are added, the degradation rate becomes positive (*k_7_* > 0) and is similar to the photoreduction process (*k_8_*). This can be attributed to the lack of h^+^, which prevents Equation (5) from generating PGO (Porous GO) and stops Equation (3) from proceeding. There is only Equation (6) which induced reduction and OH radicals continue to attack GO, making the whole system evolution similar to direct photolysis. Detailed fitting data can be found in Table S1 (Supporting Information).

### 2.4 The Role of Photoreduction for GO Photolysis

**Figure 6:**
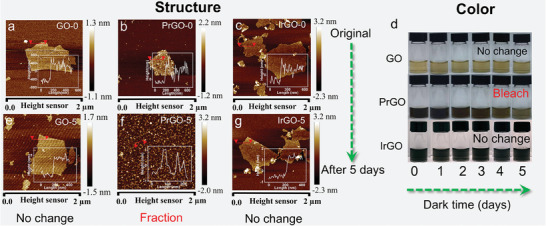
Different GO optical and structural changes in the dark environment with H2O2 for 5‐day durations. a–c) AFM images of GO, PrGO, IrGO. d) digital photos showing the color change of GO solution with different structures in the presence of H2O2 and dark conditions during 5 days. e–g) AFM images of GO, PrGO, and IrGO, after five days.

To verify this hypothesis, we comparatively studied the structural changes of the original GO, porous reduction GO, and the intact GO after photoreduction with h^+^ scavengers (EDTA) (GO, PrGO, IrGO) under dark conditions. As shown in **Figure** **6**a–c, the original GO showed an intact layer without any pores, the surface of PrGO after photoreduction has abundant pores with a diameter at ≈20 nm, while the GO after photoreduction with EDTA did not from further pore structures due to the quenching of h^+^.

We added an equal amount of H_2_O_2_ to these three types of GO and then placed them in a dark environment to exclude the influence of OH (Figure S9a, Supporting Information). Figure 6d shows the color changes of the above samples over time. Original GO and intact rGO colors did not change over a 5‐days period, while the porous rGO gradually bleached. Subsequently, Figure 6e,f shows the morphology of the corresponding products after five days in the hydrogen peroxide and dark environments. Among the three GO structures, only PrGO transformed into Nano‐GO, while the other two intact GO layers did not show significant changes. The PL spectra also confirmed this point, and only the PrGO solution produces a PL emission peak at ≈500 nm after five days (Figure S9b, Supporting Information). This slight red shift in PL spectra is due to the absence of OH radicals. The Nano‐GO formed is larger than that generated by indirect photolysis. Based on the quantum size effect, larger GQDs could exhibit a red shift in PL emissions. The results from Raman spectra also confirmed the transformation of three types of GO. IrGO and GO still show a significant D and G band after exposure to H_2_O_2_ and five days in a dark environment, while PrGO lost its characteristic D and G band (Figure S9c, Supporting Information).

This indicates that the porous structure of GO initiated by h^+^ is the key step in the fragmentation in photolysis, while H_2_O_2_ could induce the breakdown of PrGO flakes without generating OH.

Interestingly, we recorded the changes in aqueous dissolved OC (organic carbon) concentration during the PrGO bleach process, and the results are shown in Figure S10 (Supporting Information). It only decreases within one day and then keeps at a relatively stable value, which is different from the rapid decrease with the presence of OH^35^. Our opinion is that H_2_O_2_ oxidizes PrGO and then converts it into Nano‐GO, losing some organic carbon. But in the absence of OH, GO cannot be completely degraded into CO_2_, so the dissolved OC (organic carbon) concentration value remains unchanged for the rest of time. This evidence is sufficient to demonstrate that the fragmentation of GO layers in indirect photolysis is not affected by OH, mainly from photoreduction products reacting with H_2_O_2_.

**Figure 7:**
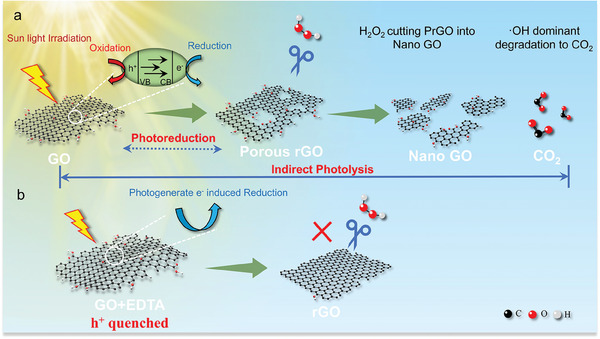
The mechanism of GO structure changes in indirect phototransformation. a) Indirect photolysis process with two distinct steps. b) Indirect photolysis process with EDTA.

Based on the above experimental results, we proposed the complete process of GO indirect photolysis illustrated in **Figure** **7**a. First, sunlight irradiation induced the transition of the GO valence band electron to the conduction band, generating a hole and a free electron. Then, free electrons combined with OFGs caused reduction and restored *π*–*π* conjugated region. The h^+^ oxidized defect carbon to CO_2_ creating a porous structure on the GO surface. In the early stage of photolysis, photoreduction predominates, and the main products are PrGO. Finally, PrGO reacts with H_2_O_2_ and OH, where H_2_O_2_ primarily induces PrGO fragmentations into Nano‐GO, while OH leads to further degradation into CO_2_.

As illustrated in Figure 7b, upon quenching the holes in photoreduction, only the electron dominates the reduction, GO is reduced into rGO with the intact surface. Then rGO no longer reacts with H_2_O_2_ and is broken into Nano‐GO. Therefore, the generation of h^+^ in photoreduction is an essential prerequisite for photolysis.

## 3 Conclusion

In summary, we investigated the effect of photoreduction in indirect photolysis of an aqueous GO solution. Our results indicate that the indirect photolysis pathway could be divided into two distinct stages. First, sunlight induces photoreduction on the GO surface, leading to the formation of PrGO. Then, H_2_O_2_ oxidizes PrGO flakes into Nano‐GO, and finally, the Nano GO is transformed into CO_2_ assisted by the OH radicals. It should be noted that photoreduction‐generated porous structure in early photolysis is a crucial premise for fragmentation and degradation. The intact GO flakes without a porous structure cannot be broken by H_2_O_2_ and OH radicals. This is the first time we provide a deep insight into the mechanism of the indirect photolysis pathway and the committed photoreduction determining the layer structure change of GO solution under irradiation. This result might be useful for designing sustainable graphene nanomaterials and enhancing GO stability. It is important to note that h^+^ scavengers could affect Advanced Oxide Process degradation GO‐related materials. This practice will provide a better understanding of GO phototransformation processes and related mechanisms.

## 4 Experimental Section

Reagents, preparation, characterizations, and methods are available in Supporting Information.

## Conflict of Interest

The authors declare no conflict of interest.

## Author Contributions

Y. Y. and N Z. contribute equally to this work. Y. Y. and N. Z. conceived and designed the research, writing original draft, investigation, data analysis, editing. S. C. performed TEM, Raman characterizations. C. M. prepared graphite oxide, data analysis. W. C. supervised the work and data analysis, Supervision. G. C. performed funding acquisition, review and editing, project administration. These authors jointly supervised this work W. C. and G. C.

## Supporting information



Supporting Information

## Data Availability

The data that support the findings of this study are available from the corresponding author upon reasonable request.
