# Supplementary material for: Toward a New Understanding of Graphene Oxide Photolysis: The Role of Photoreduction in Degradation Pathway
Source: Adv Sci (Weinh). 2025 Feb 3;12(12):2414716. doi: 10.1002/advs.202414716 (PMC11947990; doi:10.1002/advs.202414716)
Supplement: Supplementary file 1 — Supporting Information [file ADVS-12-2414716-s001.docx]

Supporting Information

Toward a New Understanding of Graphene Oxide Photolysis: The Role of Photoreduction in Degradation Pathway

Yuchen Yang, Nanzhi Zheng, Chen Ma, Silong Chen, Wenhua Chen* Guohua Chen*

Materials

Graphite powder (8000 mesh) was purchased from (Jinda technology Co., Ltd. Shenzhen, China). Sulfuric acid (H_2_SO_4_), potassium permanganate (KMnO4), hydrogen peroxide (H_2_O_2_), 2-propanol (IPA), Ethylenediaminetetraacetic acid (EDTA), were purchased from Sinopharm Chemical Reagent Co., Ltd. 5, 5-dimethyl-1-pyrroline N-oxide (DMPO), and 2,2,6,6-Tetramethylpiperidoxyl (TEMPO), were purchased from Sigma-Aldrich. All reagents were used without further purification.

Preparation of GO

GO was prepared by the modified Hummers method. Briefly, graphite (2.0 g) and H_2_SO_4_ (98%) (100 mL) were stirred in an ice bath for 0.5 h, and KMnO_4_ (12 g) was slowly added with stirring and maintaining the whole procedure below 15 °C. After removing the ice bath, the mixture was transferred into a water bath, which was heated to 45 ℃ for 2 h, and further DI water (150 mL) was added slowly, and the reaction was heated to 90 ℃ for 0.5 h. When the temperature was cooled to 30 ℃, H_2_O_2_ (30%) (15 mL) was added to give an orange-yellow solution. Then the solution was settled for 24 h and the upper layer was removed by centrifuging in DI water until the upper layer pH=7. Finally, the as-synthesized GO nanosheets were dried to solid by using a freeze-drying system.

Irradiation and sample preparation

The sunlight experiments were carried out in PCX-50C (Beijing Perfectlight Technology Co., Ltd.) equipped with an LED light source (200 mW cm^-2^). The light source wavelength is 380-780 nm to simulate solar irradiation.

GO solution as 1 mg mL^-1^ dispersion in pure water, H_2_O_2_ (100 mM, 10.2 mL) and GO solution (4 mL) was added in reaction vessels, and then diluted with pure water to 40 mL. The samples were not pH-buffered, and the initial pH was 4.0. In the quenching experiment, IPA (200 mM, 15.3 mL) was added as the • OH scavenger, and EDTA (0.5 mM, 0.146 g) was added as the h^+^ scavenger into the reaction vessels.

Intact reduction GO (IrGO), PrGO, and GO were all 0.1 mg mL^-1^ for the control experiment. different GO solutions (4 ml) and H_2_O_2_ (10 mM, 1.02 mL) were added and placed in a dark environment. Digital photos were taken every 24 h for observation.

Characterization

The transmission electron microscopy (TEM) measurement of GO and phototransformation samples was conducted using a FEI Talos F200X G2. For pristine GO and Porous rGO, we used micro grid membrance. For Nano-GO, due to its small size, we used ultrathin carbon film. Physical dimensions of GO before and after photoreaction were imaged by Atomic Force Microscopy (AFM) using Bruker Dimension ICON. GO solution is diluted and dry on mica sheet for AFM test. The AFM measurement mode is Scanasyst mode (Peakforce and Parameters Auto Adjustment). FT-IR spectra were acquired using a Thermo Scientific Nicolet iS 50. A Thermo Fisher Evolution 201 UV-visible spectrophotometer followed the change in absorbance. A 1 cm light path quartz cuvette was used with the spectrophotometer. The bonding configurations of samples were studied using an X-ray photoelectron spectroscopy (XPS, Thermo Fisher ESCALAB 250XI) with a monochromatic Al K-alpha radiation anode target and chamber pressure of 5 × 10^-10^ Torr. Aqueous dissolved OC concentrations were quantitated by a Shimadzu TOC-L CPH. Raman spectra were recorded on a Renishaw inVia Raman spectrophotometer, in which GO samples air-dried on silicon wafer were excited by a 532 nm laser beam. Fluorescence of GO and photoproducts was characterized by a Thermo Fisher lumina with a 1 cm light path quartz cuvette. The active species were detected via Electron paramagnetic resonance (ESR, Bruker EMX PLUS) using DMPO and TEMPO as the spin traps.

Statistical Analysis

To absorbance data, every sample is set three parallel experiments to ensure authentic and added error bar.


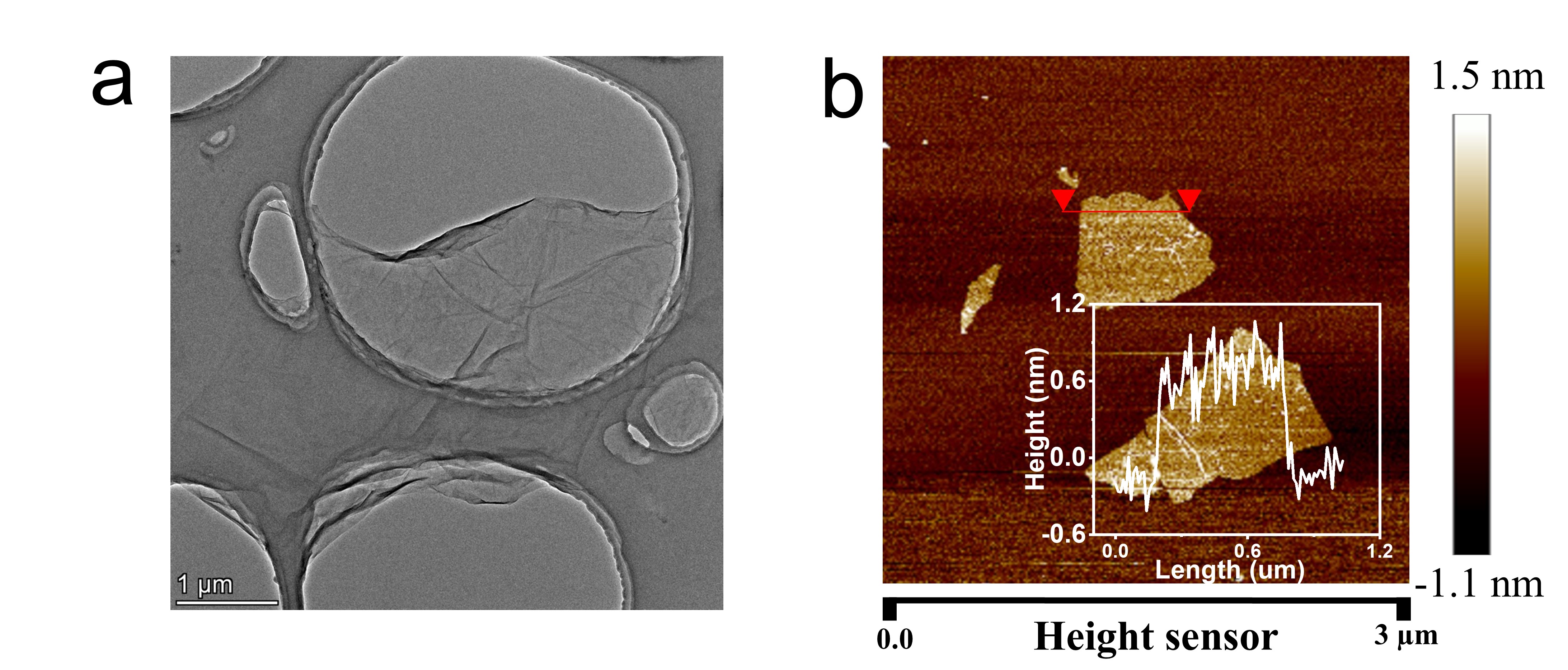


Figure S1. GO structure. a) TEM image of GO. b) AFM image of GO.


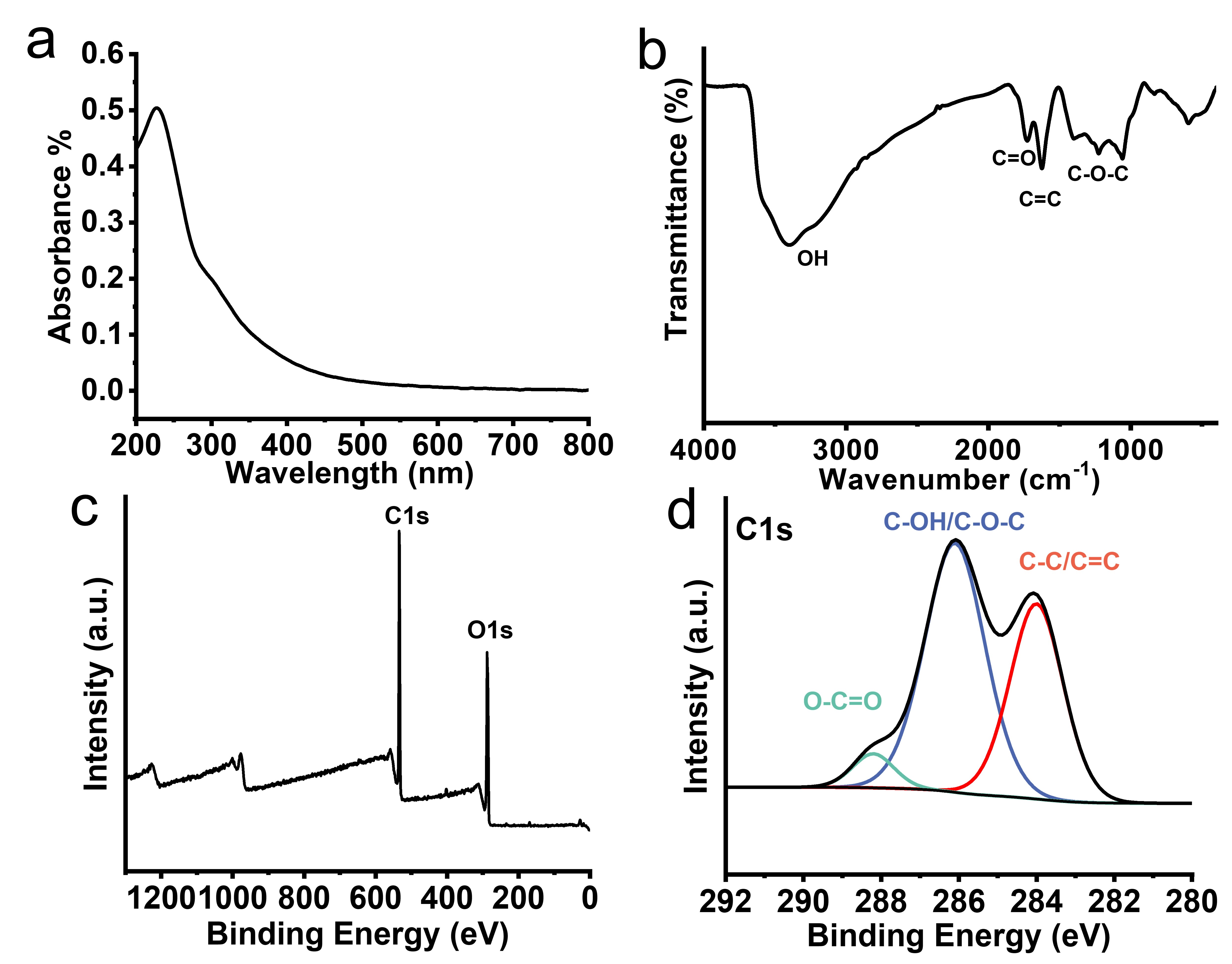


Figure S2. Optical and OFGs characterize of GO. a) the UV-visible absorbance of GO solution. b) FT-IR spectra of GO. c) XPS spectra of GO. d) XPS C1s spectra of GO.


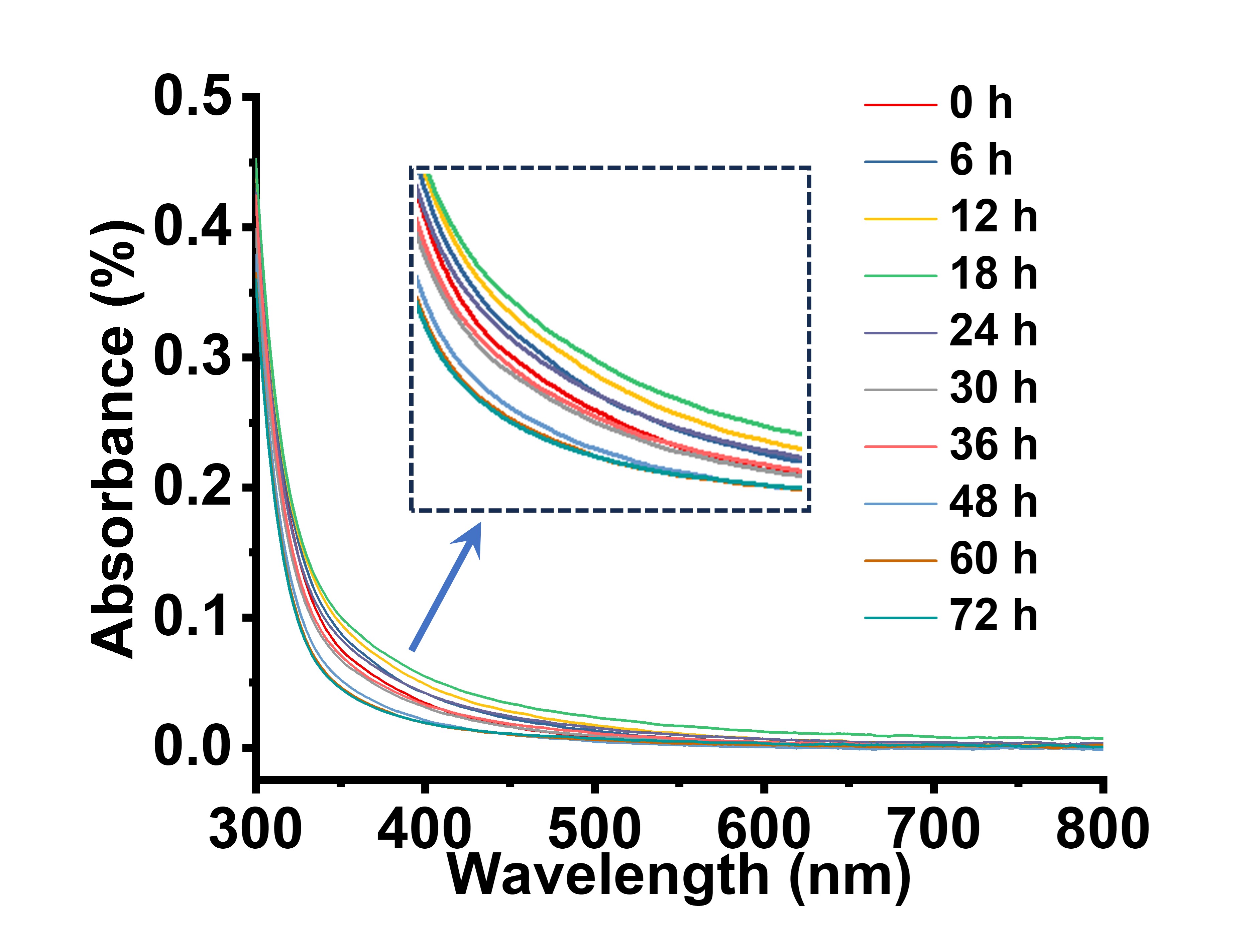


Figure S3. UV-visible absorbance at 300-800 nm of GO sample during indirect photolysis. the irradiation time: red, 0 h; blue, 6 h; yellow, 12 h; green, 18 h; purple, 24 h; gray, 30 h; pale red, 36 h; pale blue, 48 h; brown, 60 h; blue green, 72 h.


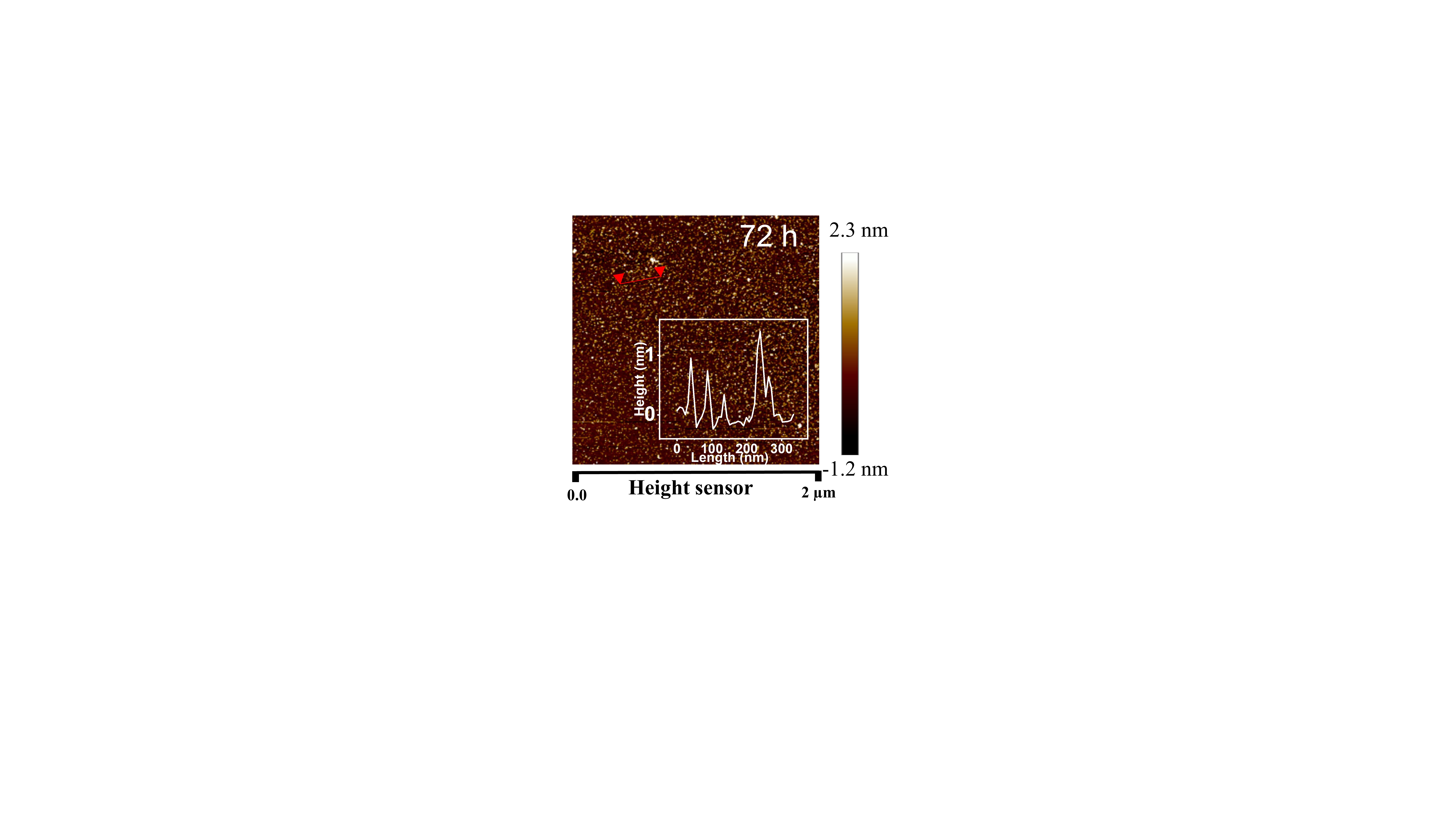


Figure S4. AFM image of GO sample at 72 h in indirect photolysis.


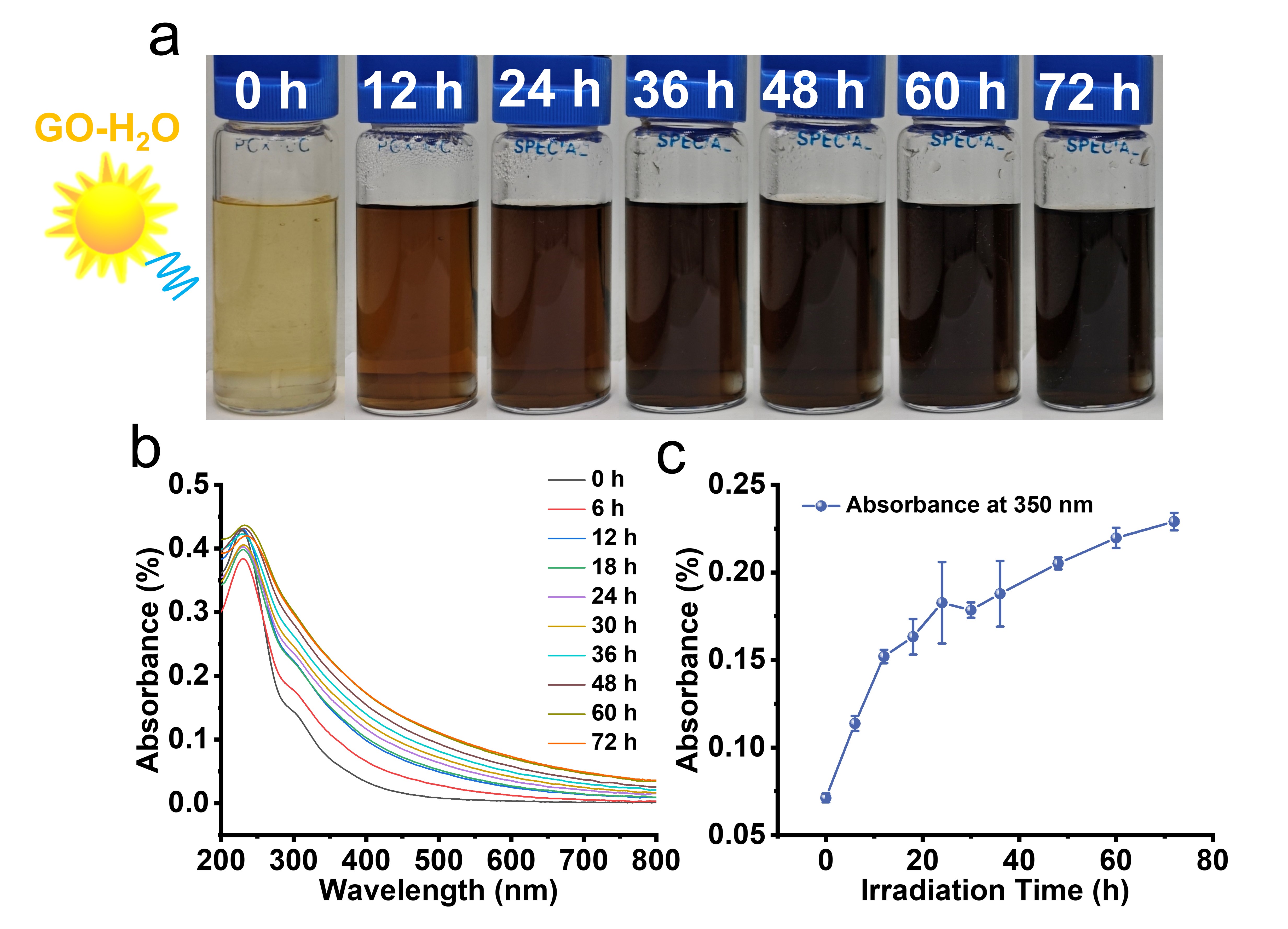


Figure S5. The evolution in direct photolysis of GO. a) color changes. b) absorbance changes. c) absorbance changes at 350 nm.


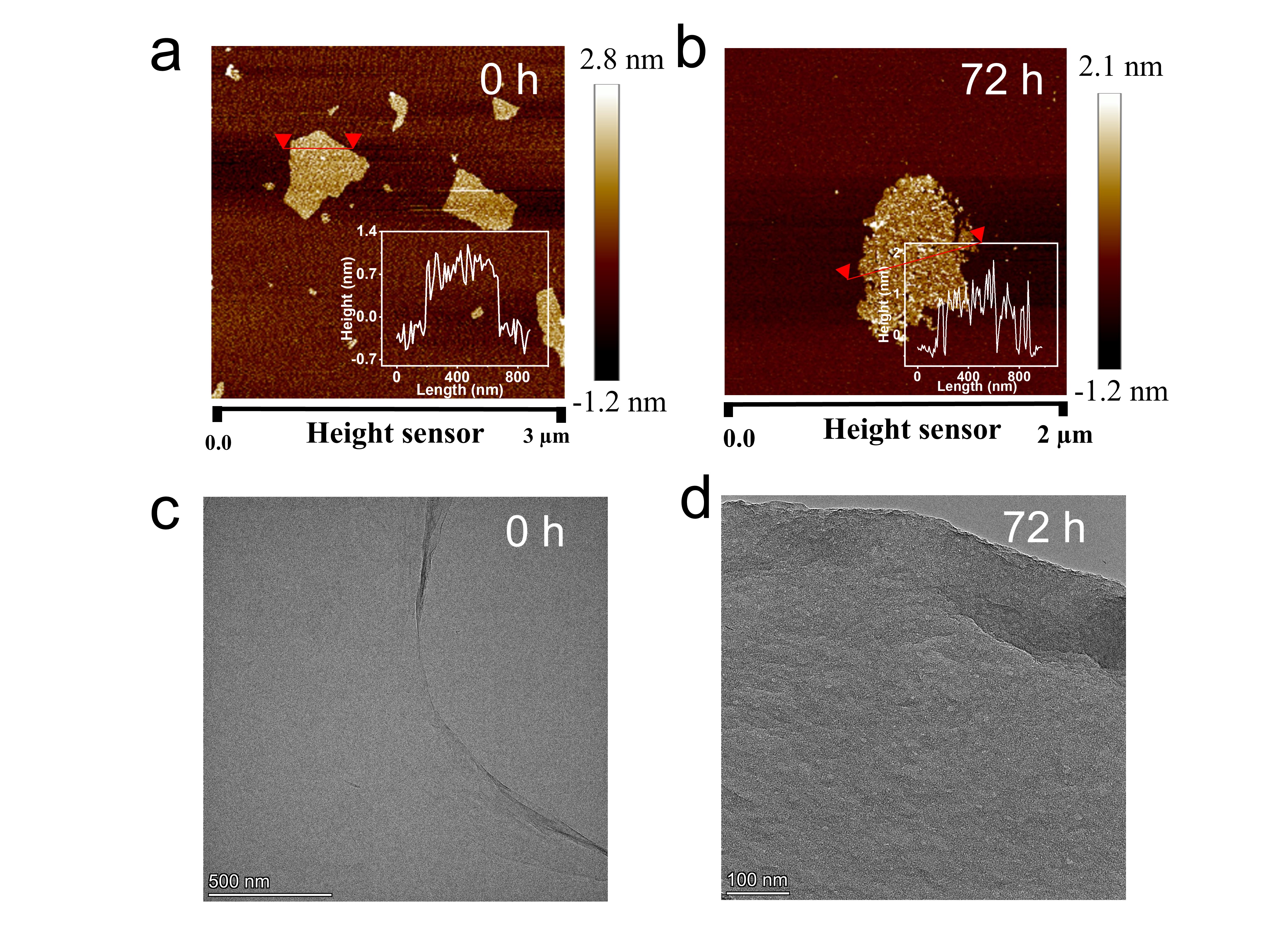


Figure S6. AFM/TEM images of GO and direct photolysis GO. a,c) pristine GO. b,d) direct photolysis GO.


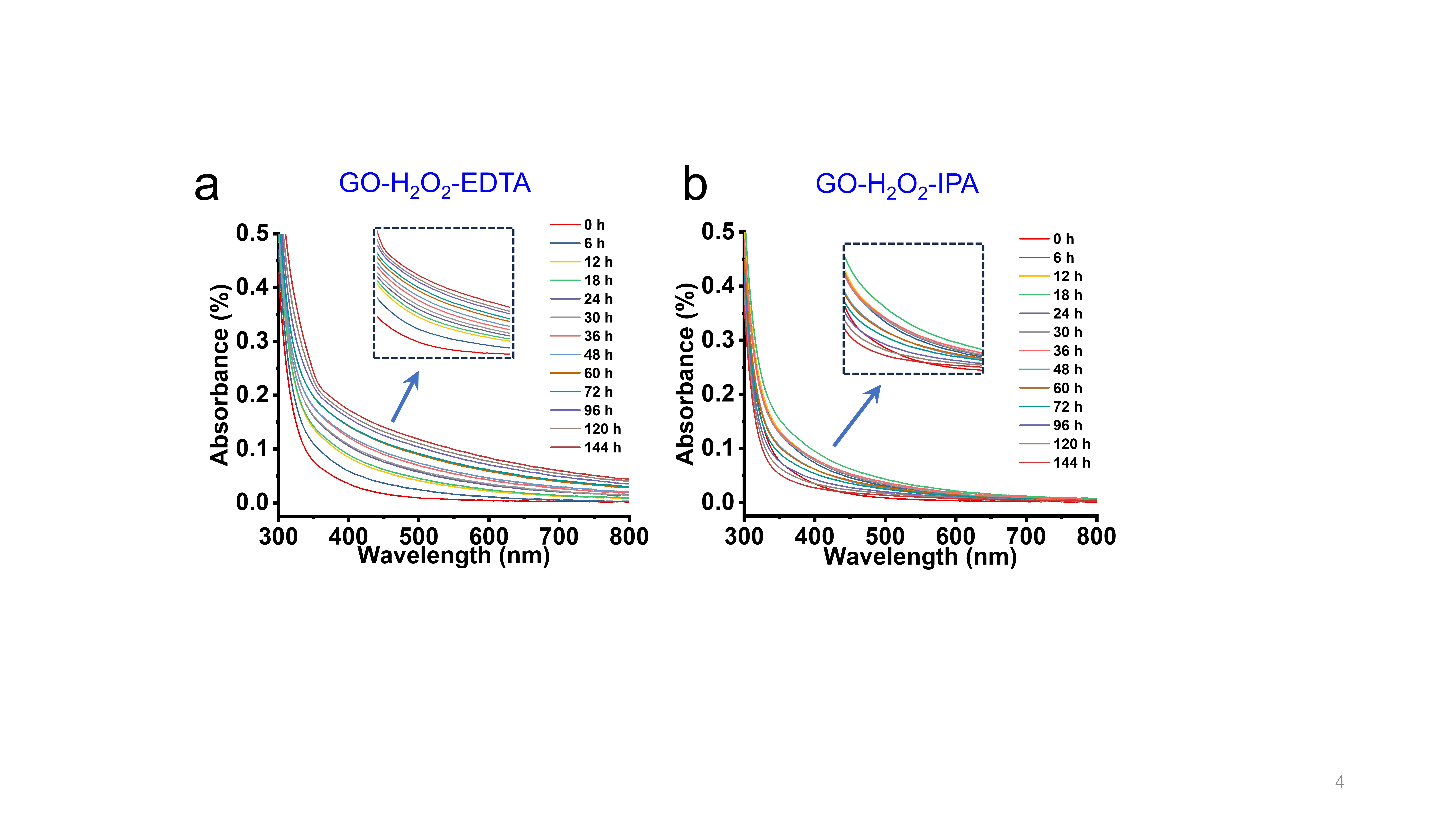


Figure S7. Changes in the UV-visible absorbance for GO indirect photolysis with scavengers. a) EDTA, the irradiation time: red, 0 h; deep blue, 6 h; yellow, 12 h; green, 18 h; purple, 24 h; gray, 30 h; pale red, 36 h; pale blue, 48 h; brown, 60 h; blue-green, 72 h; bule purple, 96h; dark gray, 120h; brown red 144h. b) red, 0 h; deep blue, 6 h; yellow, 12 h; green, 18 h; purple, 24 h; gray, 30 h; pale red, 36 h; pale blue, 48 h; brown, 60 h; blue-green, 72 h; bule purple, 96h; dark gray, 120h; brown red 144h.


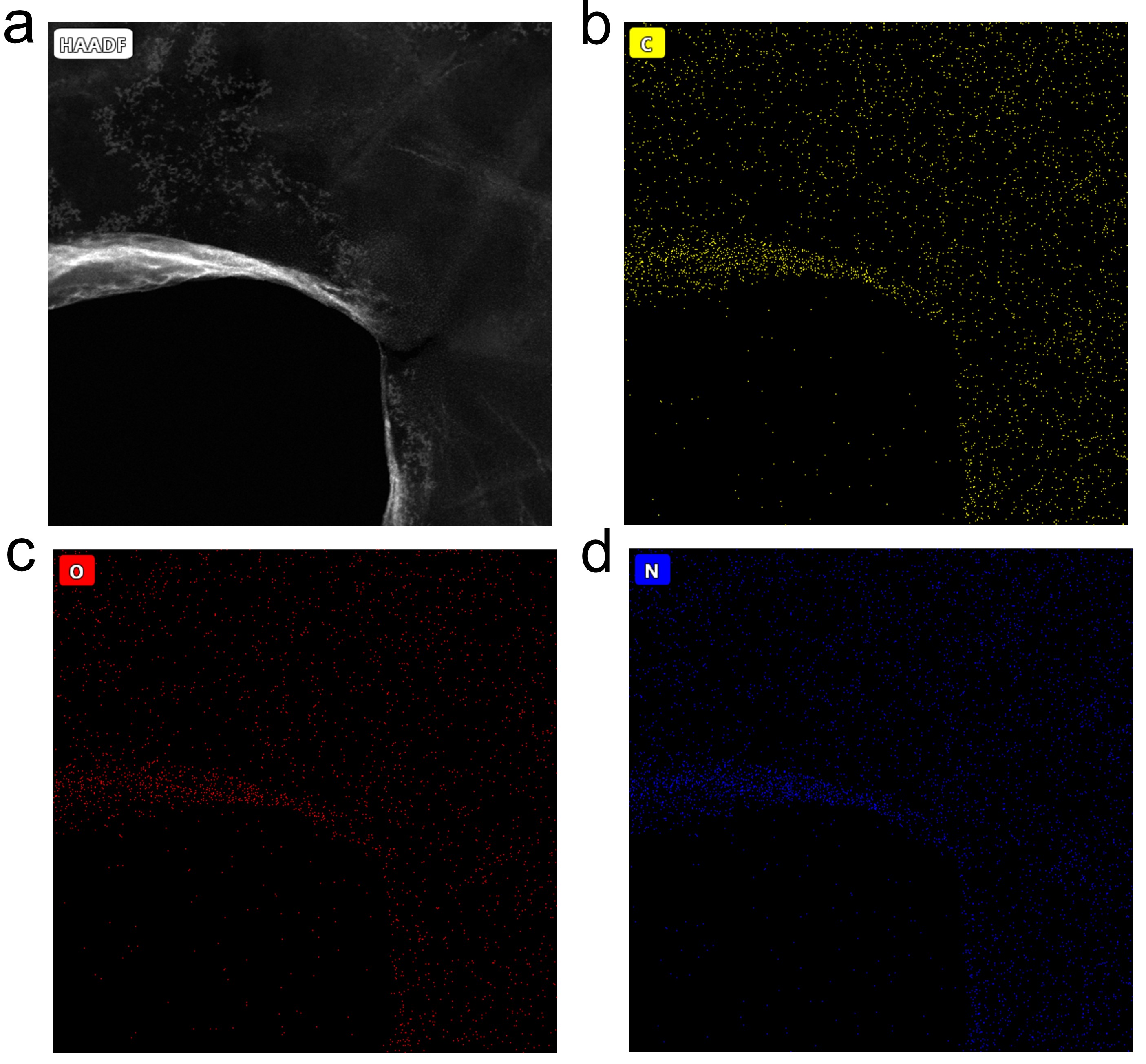


**Figure S8. EDS mapping Signal of EDTA/GO/H_2_O_2_. a)** HAADF images**. b)** carbon. **c)** oxygen. **d)** nitrogen.


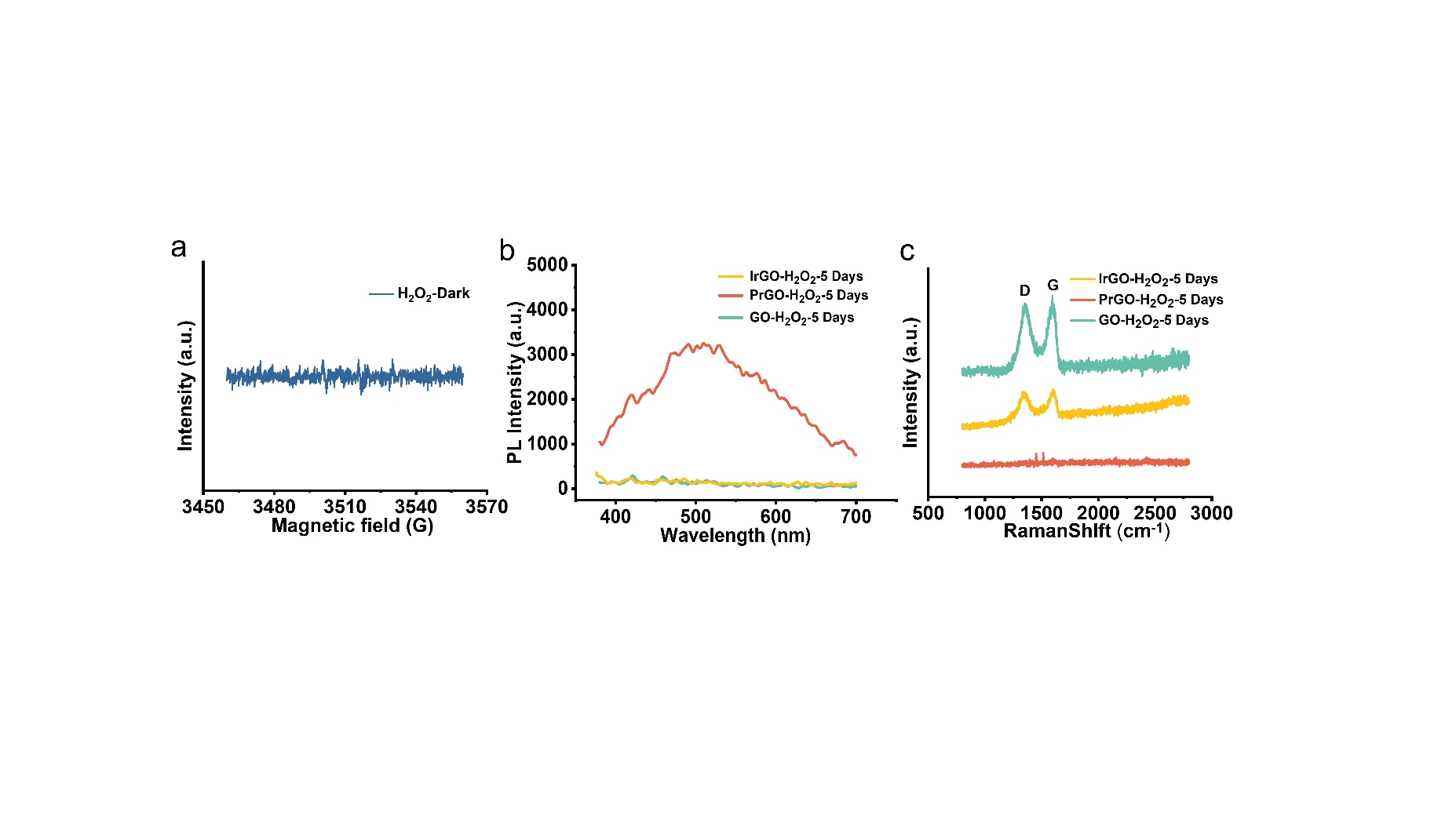


Figure S9. Dark condition ESR signal of DMPO-OH and different type GO photoluminescence patterns and Raman results. a) ESR signal of DMPO-OH in dark environment. b) PL intensity of different structure GO sample after five days, Ex wavelength at 365 nm. c) Raman results of three kinds of GO sample after five days.


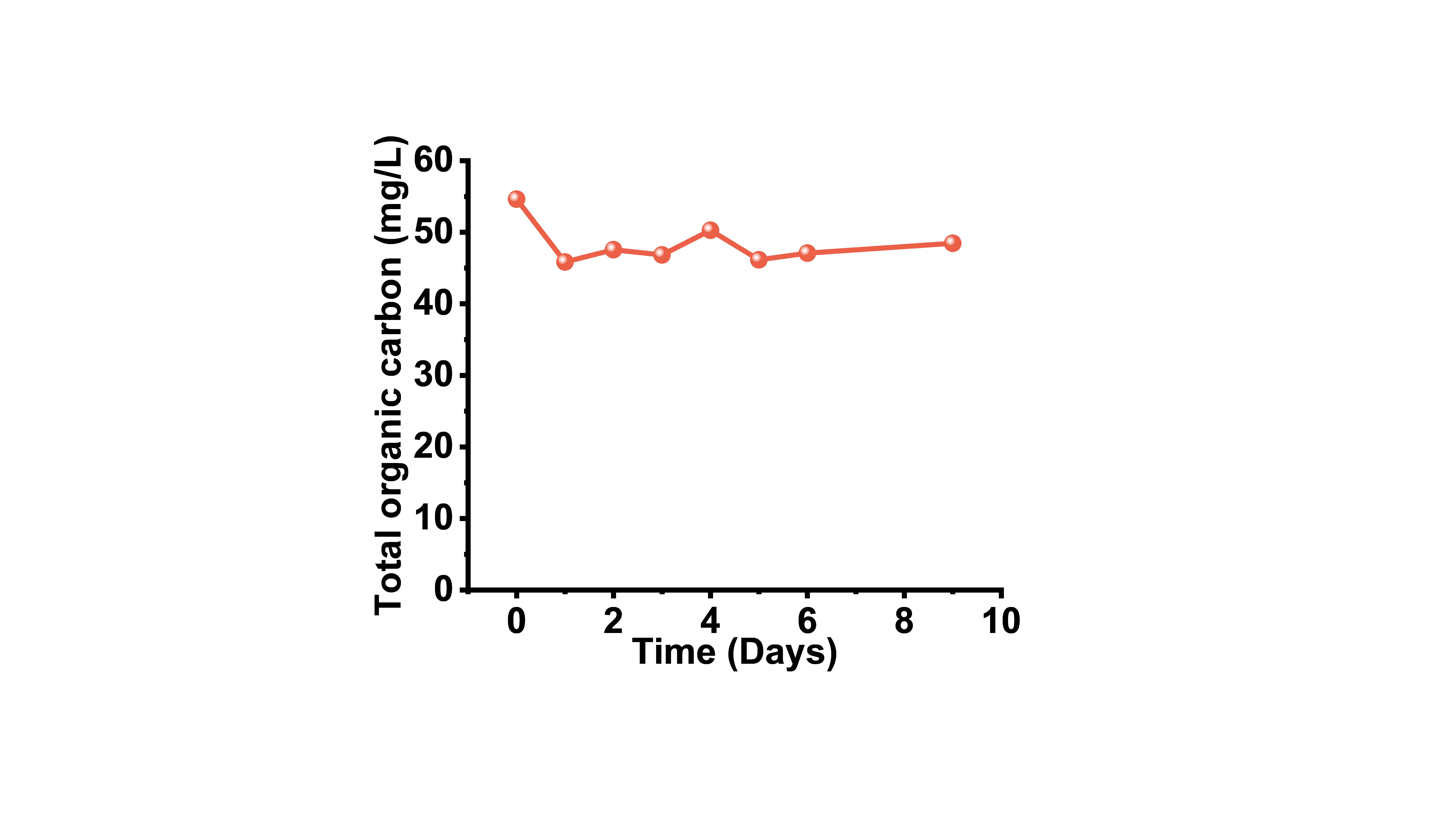


Figure S10. Aqueous dissolved OC concentrations changes of sample (PrGO) during dark time and H_2_O_2_.

**Table S1. Fitting data of kinetics analysis.**

| **System**  **Stage** | **GO/H_2_O** | **GO/H_2_O_2_** | **IPA/GO/H_2_O_2_** | **EDTA/GO/H_2_O_2_** |
| --- | --- | --- | --- | --- |
| **Early stage** | y=0.00632x+0.07263  R^2^=0.981 *k_4_*=0.00632 | y=0.00189x+0.08511  R^2^=0.974 *k_1_*=0.00189 | y=0.00401x+0.08065  R^2^=0.975 *k_2_*=0.00401 | y=0.00503x+0.07761  R^2^=0.974 *k_3_*=0.00503 |
| **Later stage** | y=0.00126x+0.1424  R^2^=0.969 *k_8_*=0.00126 | y=-0.00142x+0.1288  R^2^=0.720 *k_5_*=-0.00142 | y=-0.000926x+0.01607  R^2^=0.941 *k_6_*=-0.000926 | y=0.00109x+0.131  R^2^=0.954 *k_7_*=0.00109 |
